# Continuity of care for children with anorexia nervosa in the Netherlands: a modular perspective

**DOI:** 10.1007/s00431-024-05497-4

**Published:** 2024-03-12

**Authors:** A. J. Lennips, V. J. T. Peters, B. R. Meijboom, A. C. Nissen, J. E. H. Bunt

**Affiliations:** 1 Department of Health Services Management & Organization, Erasmus School of Health Policy & Management, Erasmus University Rotterdam, Rotterdam, the Netherlands; 2 Department of Information Systems and Operations Management, Tilburg School of Economics and Management, Tilburg University, Tilburg, the Netherlands; 3 Department of Tranzo, Tilburg University, Tilburg, the Netherlands; 4 Department of Pediatrics, Elisabeth-TweeSteden Ziekenhuis, Tilburg, the Netherlands

**Keywords:** Modularity, Continuity of care, Anorexia nervosa

## Abstract

Care provision for children with anorexia nervosa is provided by outpatient care teams in hospitals, but the way these teams are organized differs per hospital and hampers the continuity of care. The aim of this study is to explore the organization and continuity of care for children with anorexia nervosa in the Netherlands by using a modular perspective.

We conducted a qualitative, exploratory case study and took the healthcare provision for children with anorexia nervosa, provided by outpatient care teams, as our case. We conducted nine interviews with healthcare professionals involved in outpatient care teams from six hospitals. A thematic analysis was used to analyze the data.

The modular perspective offered insights into the work practices and working methods of outpatient care teams. We were able to identify modules (i.e. the separate consultations with the various professionals), and components (i.e. elements of these consultations). In addition, communication mechanisms (interfaces) were identified to facilitate information flow and coordination among healthcare professionals. Our modular perspective revealed gaps and overlap in outpatient care provision, consequently providing opportunities to deal with unnecessary duplications and blind spots.

*   Conclusion*: A modular perspective can be applied to explore the organization of outpatient care provision for children with anorexia nervosa. We specifically highlight gaps and overlap in healthcare provision, which in turn leads to recommendations on how to support the three essential parts of continuity of care: informational continuity, relational continuity, and management continuity.

**Table:** 

**What is Known:** *• Care provision for children with anorexia nervosa requires a network of health care professionals from different organizations, as a result the organization and provision of care faces challenges.*	
**What is New:** *• Modular care provision sheds light on the complexity and organization of outpatient care provision and supports the three dimensions of continuity of care as experienced by children with anorexia nervosa and their parents/caregivers.*

**What is Known:**

*• Care provision for children with anorexia nervosa requires a network of health care professionals from different organizations, as a result the organization and provision of care faces challenges.*

**What is New:**

*• Modular care provision sheds light on the complexity and organization of outpatient care provision and supports the three dimensions of continuity of care as experienced by children with anorexia nervosa and their parents/caregivers.*

## Introduction

The number of children that suffer from complex medical conditions is steadily increasing across the globe [1]. When treatment of these complex medical conditions requires multiple healthcare professionals from different organizations, the continuity of care (COC) becomes problematic [2]. The concept of COC is defined as the degree to which patients perceive their received care as coherent, coordinated, and unduplicated [3] and consists of three essential parts: information continuity, relational continuity, and management continuity [4]. Previous research shows that children with complex medical conditions and parents/caregivers experience dissatisfaction regarding COC throughout their episodes of care [2]. This observation holds true for children with anorexia nervosa (AN) which is a complex psychiatric disorder with potential severe somatic and psychosocial consequences. Children with AN require a network of professionals from different organizations [5, 6] and, as a result, the organization and provision of care for children with AN face challenges [7]. Previous research outlines the challenges associated with the organization of outpatient care provision for children with AN, including the need for integrated and person-centered multidisciplinary approaches [7], dissatisfaction with care professionals [8], a lack of communication and information sharing among care professionals [5], and endangered COC [6]. COC is endangered when the organization of care is complex, which can lead to overlaps and gaps in treatment and, consequently, poor health outcomes for children with AN.

Theoretically, the modularity concept can help healthcare professionals to obtain insight into the complex organization of outpatient care provision for children with AN. Modularity is increasingly recognized in healthcare as a means to obtain insight into the provision of complex care [9–12]. It can be used to reduce complexity by its ability to decompose healthcare services into modules and components. Modules are relatively independent parts of healthcare provision with a specific function. Components are elements of healthcare provision that are offered as part of a module; they have a distinct function but cannot function independently [9]. These individual modules and components can then be used, omitted or postponed (mix-and-match) to meet individual patient needs [11]. Communication mechanisms should be in place that ensure that components and modules can be used to meet individual needs and form a coherent whole; these are called interfaces [10]. Previous research on care provision for children with complex chronic care needs show that applying a modular perspective leads to insights into the work practices of professionals and working methods of multidisciplinary care teams. This increases transparency for healthcare professionals, children and parents/caregivers [9, 12]. In addition, the modular perspective revealed gaps and overlaps in healthcare provision and provided opportunities to deal with unnecessary duplications and blind spots [9, 12]. This awareness increased coordination of care and, consequently support COC as experienced by children, their parents/caregivers, and professionals. However, little is known about the potential of modularity for children with more acute complex care needs like children with AN.

In the Netherlands, pediatricians provide care for children with AN in an outpatient care team, in collaboration with other healthcare professionals. These outpatient care teams are not explicitly designed in a modular way. Although there is a general guideline for the provision of care for children with an eating disorder, developed under the auspices of the Dutch Pediatric Association [13], it does not provide a clear direction on how to organize outpatient care provision for children with AN. As a result, the work practices and working methods differ from hospital to hospital and best practices for the organization of outpatient care provision for these children have not been identified yet.

The aim of this study is to explore the organization of care for children with AN in the Netherlands by using a modular perspective and to examine how this perspective might support the COC.

## Methods

### Study design

The consolidated criteria for reporting qualitative research (COREQ) [14] were used as a guideline for study design, data collection, and data analysis for this qualitative, exploratory study involving semi-structured interviews with healthcare professionals providing outpatient care for children with AN in the Netherlands (Appendix 1). Locally available documentation on the organization of care was retrieved from each outpatient care team (e.g. local guidelines, documentation of local collaborations, documents on implementation of national guidelines). The information from the local documents was incorporated in the interpretation and analysis of the data. The Ethics Review Board of Tilburg University decided that ethics approval was not required because no patients were involved in this study.

### Recruitment and sample

We contacted pediatricians of 26 large hospitals in the Netherlands providing outpatient care for children with AN. In six hospitals, pediatricians expressed their interest to participate in our study. We conducted interviews with coordinators (in all cases the pediatrician) from the six outpatient care teams. We deliberately chose to interview the coordinators of these outpatient care teams as they had expertise and experience in the field of healthcare provision for children with AN and knowledge on the work practices and working methods of each respective outpatient care team. These six outpatient care teams demonstrated variety in involved healthcare professionals and geographic locations, leading to a comprehensive view on outpatient care provision for children with AN. We used purposive sampling strategy and conducted interviews with seven pediatricians from the six selected hospitals involved in outpatient care provision for children with AN (Table 1). The participating pediatricians suggested to interview representatives from Dutch mental healthcare institutes as well, given their experience with and expertise on psychiatric care provision for these children. Snowball sampling was used to conduct two additional interviews with representatives from Dutch mental healthcare institutes (Table 1). All representatives served as a (former) member of an outpatient care team.

**Table 1:** Respondent characteristics

Respondent	Organization	Profession	Years of experience	Gender
Respondent A	Hospital A	Pediatrician	20 years	Male
Respondent B	Hospital B	Pediatrician	15 years	Female
Respondent C	Hospital C	Pediatrician	2 years	Male
Respondent D	Hospital D	Pediatrician	9 years	Female
Respondent E	Hospital D	Pediatrician	11 years	Female
Respondent F	Hospital E	Pediatrician	20 years	Female
Respondent G	Hospital F	Pediatrician	20 years	Female
Respondent H	Mental healthcare institute	Operations manager	5 years	Female
Respondent I	Mental healthcare institute	Nursing specialist	12 years	Male

### Data collection

We conducted semi-structured interviews either in person, by phone, or via Zoom in May 2022. We allowed for phone and Zoom interviews given the Covid-19 restrictions at hand. An interview topic guide, based on scientific literature [4, 9] and expert knowledge, was used for the interviews (Appendix 2). Oral approval for participation in the study was received from each participant; each interview was audio-recorded and transcribed verbatim. Participants were asked to review their own transcript to improve the reliability of our interpretations. This was done to check for accuracy and resonance with the experiences of the participants [15]. Participants did not change any transcript. We also collected local documents (e.g. medical protocols, national guidelines, screening forms, work schemes) from each outpatient care team; these documents were discussed with the respective participant. This information was used to interpret the interview data from the respective participant and gave additional insight in the particular organization of care of each team.

### Data analysis

The final data consisted of transcripts of the interviews and documentation. The different types of data were complementary to each other: the interviews helped us to acquire information on the professional’s perspective on care provision, whereas the collected documents provided information with regard to the composition and working methods of the outpatient care teams. A coding reliability approach to thematic analysis was carried out [16] in which theory does inform the early development of themes and codes. We followed this approach because it allowed us to recognize modularity and COC in outpatient care provision for children with AN. We used theory on modularity and COC for the early development of the themes. This approach involves the use of a structured codebook in which codes are predetermined (Appendix 3). The participants did not express themselves in modularity terms, but instead we used modularity as a perspective that guided interpretation of the data. This kind of modular interpretation of research contexts has been applied frequently in the existing healthcare modularity literature [e.g. 9,10,12]. By combining the information from the interviews and documentation, we were able to describe and interpret the practices of the outpatient care team in modular terms. For example: we used the national guideline [14] to identify elements of outpatient care provision for children with AN as components, following our used definition of components [9].

## Results

### Recognizing modularity in outpatient care provision for children with anorexia nervosa

We were able to describe the healthcare practices in modular terms based on the interview data and collected documents. During outpatient care provision for children with AN, the child meets various healthcare professionals. From a modular perspective, the individual consultations with the various professionals comprise the *modules*. Examples of modules are ‘Consultation with the Pediatrician’ and ‘Consultation with the Dietician’. To illustrate: “*We [pediatricians] do not provide the care alone, we also have the support of experienced dietitians who are involved in care provision for children with anorexia nervosa and have a great sense for these children*” *– Respondent A*.

Components are elements of healthcare provision that have a distinct function but cannot function independently: they are offered as part of the module, in our case, the consultation. The components are based on guidelines, protocols, and screening forms used in outpatient care provision for children with AN; they are offered as part of a module. The components prescribe what kind of activities are being carried out during a consultation [module]. Examples of components are ‘Nutritional needs’, ‘Somatic state’, and ‘Physical examination’. To illustrate: “*During the first visit, I always perform a blood test as standard element [component] of the consultation [module] to see, based on blood values, whether a child is suffering from not eating much. An electrocardiogram is also exemplary of an element [component] that I always perform” – Respondent B.*

**Fig. 1:**
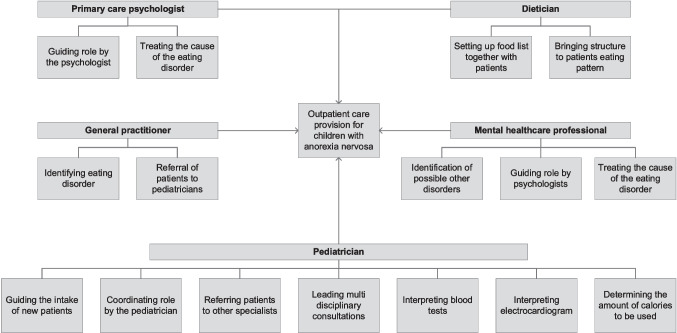
Modular composition of outpatient care provision for children with AN in Hospital A

**Fig. 2:**
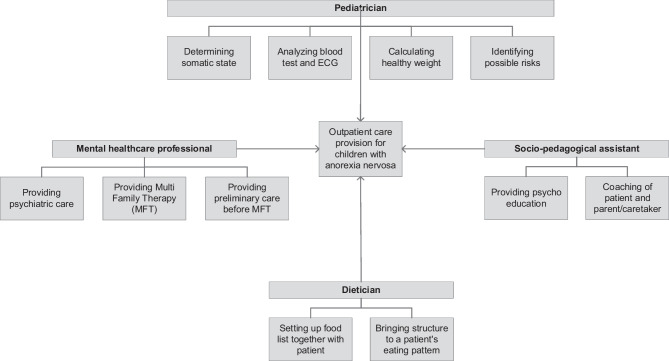
Modular composition of outpatient care provision for children with AN in Hospital B

**Fig. 3:**
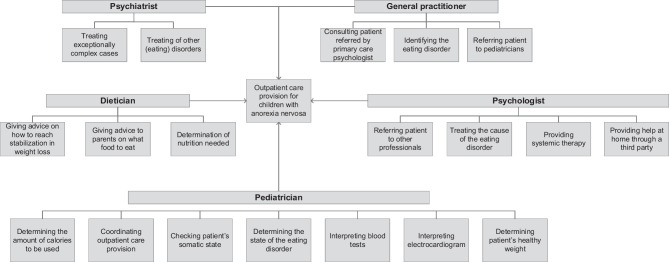
Modular composition of outpatient care provision for children with AN in Hospital C

**Fig. 4:**
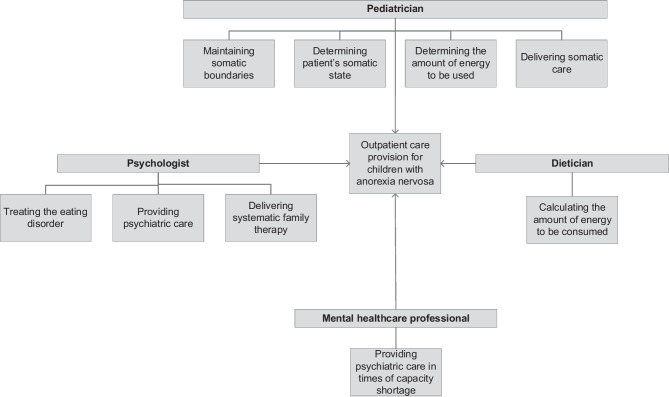
Modular composition of outpatient care provision for children with AN in Hospital D

**Fig. 5:**
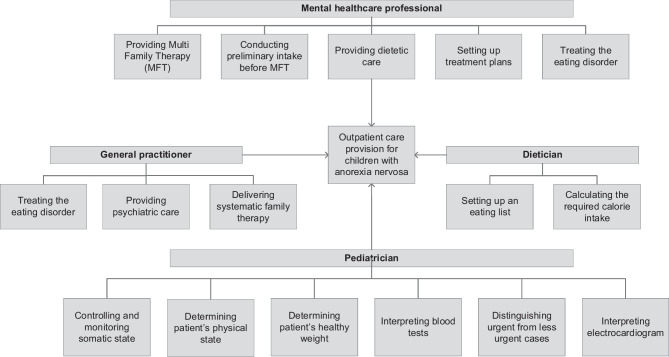
Modular composition of outpatient care provision for children with AN in Hospital E

**Fig. 6:**
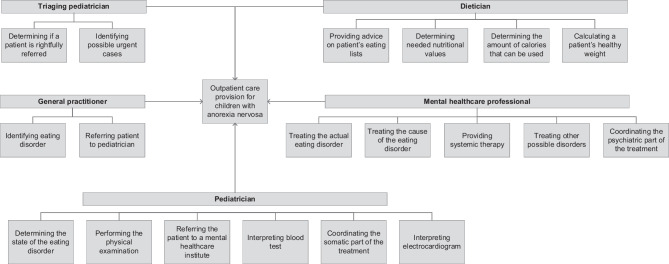
Modular composition of outpatient care provision for children with AN in Hospital F

The six hospitals studied offered four to five different modules, wherein various components were distinguished (a comprehensive picture of the modular composition of outpatient care provision for children with AN as delivered by Hospital A is provided in Fig. 1; a comprehensive picture of the modular composition of the other five participating hospitals is shown in Appendix 4 (Figs. 2, 3, 4, 5 and 6).

We also found several communication mechanisms that connected the modules (i.e. the individual consultations). Using a modular perspective, these communication mechanisms are defined as interfaces. The majority of interfaces contributed to the flow of information in outpatient care provision as they helped to manage and guide interaction between the healthcare professionals involved. Multidisciplinary meetings, referral letters, electronic health record (EHR) reports, and direct communication lines (mail, phone call, instant messaging) are examples of interfaces that guide information-flow. For example, the referral letter ensures that information about a patient’s health status is being transferred from one professional to another, both within and outside of the hospital. We also identified interfaces like protocols, guidelines, and screening forms that guide professionals’ judgment in the selection of appropriate components for each child, ensuring that outpatient care provision meets the needs of each individual child. The identified interfaces highlight the interdependency of the involved healthcare professionals in outpatient care provision for children with AN. For example, when new information becomes available about a child (e.g. evidence of abuse), the pediatrician will inform the other professionals about the new information via the EHR or a referral letter.

### Supporting continuity of care in outpatient care provision for children with anorexia nervosa

By explicitly decomposing outpatient care provision into modules, components, and interfaces, we were able to recognize how modularity can support the three essential parts of COC.

First, informational continuity is related to the availability of a child’s medical history, at all times, for each professional. Although the EHR ensures easy and quick access to a child’s medical history, not all professionals have direct access to the EHR. The professionals without access to the EHR need to actively request information from the healthcare professionals or patients and parents/caregivers, resulting in professionals sometimes lacking information. To illustrate: “*I [pediatrician] cannot access the files of the psychologists and it does matter that I know what the psychologist is doing and *vice versa*” – Respondent B*. The modular perspective explicitly highlighted this issue, since no (active) information exchange [interface] was present, for example, between the pediatrician and the psychiatrist, resulting in missed information during consultations. Actively exchanging information would ensure no information is lost, increasing efficiency of the consultation, and would result in supported informational continuity.

Second, relational continuity refers to the familiarity between professionals, children and their parents/caregivers. Respondents argued that they all try to realize relational continuity in care provision by ensuring that there is a dedicated professional for each child, resulting in lasting patient-professional relationships. For example, one pediatrician argued that seeing the same child multiple times makes consultations more effective and efficient and increases the level of trust: *“By seeing the same child, consultations could become more efficient and would not have to take long, whereas if the child visits my colleague [pediatrician] and me interchangeably, there might be redundant information exchange possible” – Respondent A*. This is especially relevant because it is quite an intense and emotional care trajectory for children and parents/caregivers, and it is important to establish levels of trust between all involved parties. However, relational continuity is at risk when healthcare professionals are temporarily absent, and the child needs to visit another healthcare professional. Respondents argued that there is no multidisciplinary workbook [interface] available that they can use to inform their fellow colleagues on the working methods in outpatient care provision for children with AN. The availability of such a workbook would improve the relational continuity of outpatient care provision.

Last, a lack of management continuity has been observed because for example, the pediatrician, general practitioner, and psychiatrist all have separate care plans, separate EHRs, and work in different organizations resulting in potentially missing or overlapping information and treatment. Management continuity refers to the extent to which the provided care is perceived as coherent, coordinated, and unduplicated. Respondents argued that it is not always clear for patient and parents/caregivers what element of care will be performed by whom: *“Although most of us [pediatricians] focus on medical care and less so on psychological or psychiatric care, we have to incorporate those elements into our consultation to obtain an overall view of the patient in front of us. This might be confusing for patients because the information might also be obtained elsewhere” – Respondent A*. Not only is this confusing for patients and parents/caregivers, but also for professionals themselves as the content of healthcare provision was not prescribed in detail for each professional, resulting in different professionals who ended up doing the same thing or not doing it at all according to a pediatrician: “*I do not have a clear overview [modules and components] of what truly happens in mental care for example, which also limits me in that sense in my healthcare provision” – Respondent F*. Having decomposed and allocated the different aspects of the professional’s work, it is possible to identify identical and missing components in care provision for children with AN, which would support management continuity.

## Discussion

Our study shows that approaching the medical domain while utilizing modularity, a concept from the domain of operations management [9–11] leads to new and valuable insights on the organization of outpatient care teams. This interdisciplinary approach indicates that a modular perspective is applicable to analyze the acute complex care needs as required in outpatient care provision for children with AN. The modular perspective allowed to perform a comparative analysis between the outpatient care teams providing care for these children and to obtain a comprehensive understanding of their working methods and work practices. The results show both similarities and differences in the organization that studied the outpatient care teams of the different hospitals which led to mutual insight for healthcare professionals involved. In all outpatient care teams, the pediatricians occupied the role of coordinator. However, the way these roles are shaped, and the content of these roles differ between teams which aligns with previous research that highlights the various roles of pediatricians in identification and management of eating disorders in children [17]. All respondents highlighted COC as one of the important issues that deserves more attention in outpatient care provision for children with AN. Our modular perspective led to increased awareness of COC for children with AN: the respondents mentioned that a considerable amount of patient information might be redundantly collected, given the scarcity of information sharing between professionals. As a result, the COC became endangered because not every professional had access to the right patient information at the right time, leading to duplication and gaps in the care provided. When interfaces are in place that ensure timely and accurate information sharing between patients, the COC can be supported [12, 18]. The modular perspective explicitly offered clues for resolving challenges regarding information collection and exchange, which is relevant whenever the information need arises for treatment of children suffering from AN. Our study shows that a modular perspective can be used to increase transparency on care provision for professionals, patients and their parents/caregivers. This is in accordance with the findings of other studies [9, 10, 12], albeit the limited availability of studies on the use of modularity in acute complex care provision.

Whereas a decent infrastructure is in place regarding the organization of pediatric care for children with AN, this is certainly not the case once individuals reach the age of 18. They are often lost as they leave pediatric care, and it is largely unclear how and where adults with AN receive healthcare later in life. This might stem from the fact that it is not clear which professional is responsible for the wellbeing of these individuals. In addition, the current health system adheres to age-based categorizations, however, conditions like AN do not adhere to these boundaries. In the Netherlands, there is currently no transition protocol available for the transition from pediatric to adult care. This compromises the COC for these young adults, despite previous research on transitional care highlighting the importance of a well-organized transition for other patient groups [19, 20]. Therefore, we recommend that future research should focus on the transition from pediatric to adult care and advocate the use of modularity to develop transition guidelines.

### Limitations and future research

First, the respondents did not a priori consider their care provision as being modular, nor did they express themselves using modularity concepts. Instead, we used modularity as a perspective that guided interpretation of the way of working of the outpatient care teams. This meant that the data had to be interpreted in modularity terms; this kind of interpretation of research contexts has been frequently applied in previous research [9, 10, 12, 18], it might have resulted in a researcher bias. Future research should focus on care provision that is designed in a modular way and would allow for comparative studies between explicit and implicit modular care provision. Second, our study solely focuses on the perspective of pediatricians. Many different care providers are involved with the care for children with AN. Although the pediatricians have an overview of the provided care, each of the other involved care providers may have different attitudes, views and knowledge on the organization of care for this patient group. Therefore, our research could be expanded to including other involved care providers (e.g. child psychiatrists) as this would maximize the expression of diversity in the sample and could bring out divergences. Last, the perspective from children and parents/caregivers had not been included which should be aimed at in future studies to gain insights into how the organization of care impacts patients’ experiences. Incorporating their view in future research is important to create a complete and nuanced picture of the provided care, especially for obtaining more knowledge on the transition from pediatric to adult care.

## Conclusion

We explored the organization of outpatient care provision for children with AN in the Netherlands given the complexity in terms of content, organization and access of care. The theoretical concept of modularity was used to reduce the inherent complexity of outpatient care provision for children with AN and to obtain insights into the work practices and working methods of outpatient care teams. This revealed gaps and overlaps, consequently addressing unnecessary duplications and blind spots in the organization of outpatient care provision. This study suggests that a modular perspective might support COC in outpatient care provision for children with AN.

## Data Availability

The raw data is not publicly available to preserve individuals.

## Appendix 1. A 32-item checklist for reporting qualitative studies (COREQ)

**Table:**

**Item**	**Description**	**Check**
**Domain 1: Research team and reflexivity**		
*Personal characteristics*		
1. Interviewer	Which author/s conducted the interviews and/or observations?	Author 1
2. Credentials	What were the researcher’s credentials?	Author 1: MSc Author 2: Dr Author 3: Prof. dr. ir Author 4: MSc Author 5: Dr
3. Occupation	What was the occupation at the time of the study?	Author 1: PhD student Author 2: Postdoc researcher Author 3: Endowed professor Author 4: Pediatrician Author 5: Pediatrician
4. Gender	Was the researcher male or female?	Author 1: Male Author 2: Male Author 3: Male Author 4: Female Author 5: Male
5. Experience and training	What experience or training did the researcher have?	Author 1: Courses on Qualitative Research Methods at Tilburg University and previous experience during thesis writing Author 2–5: A lot of experience with peer reviewed scientific output on healthcare, modularity and clinical studies
*Relationship with participants*		
6. Relationship established	Was a relationship established prior to study commencement?	A professional relationship was established with the pediatricians, based on previous collaborations, ahead of the research
7. Participant knowledge of the researcher	What did the participants know about the researcher?	The personal interest of the researchers and purpose of the study was explained before the data collection started
8. Researcher characteristics	What characteristics were reported about the researcher?	Interest in research topic, occupation, reason for research
**Domain 2: Study design**		
*Theoretical framework*		
9. Methodological orientation and Theory	What methodological orientation was stated to underpin the study?	Coding reliability approach to thematic analysis
*Participant selection*		
10. Sampling	How were participants selected?	Purposive sampling
11. Method of approach	How were participants approached?	Telephone, face-to-face, e-mail
12. Sample size	How many participants were in the study?	9
13. Non-participation	How many people refused to participate or dropped out? Reasons?	0
*Setting*		
14. Setting of data collection	Where was the data collected?	Hospital (in-person), university (via telephone),and digital (via Zoom)
15. Presence of non-participants	Was anyone else present besides the participants and researchers?	No
16. Description of sample	What are the important characteristics of the sample?	Gender, profession, role in outpatient care, years of experience
*Data collection*		
17. Interview guide	Were questions, prompts, guides provided by the authors? Was it pilot tested?	The topic list is added to the manuscript; the topic list was pilot tested and discussed within the study team
18. Repeat interviews	Were repeat interviews carried out? If yes, how many?	No repeat interviews were carried out
19. Audio recording	Did the research use audio recording to collect the data?	Yes
20. Field notes	Were field notes made during and/or after the interview or observation?	Yes
21. Duration	What was the duration of the interviews or observation?	Interviews: 25–45 min
22. Data saturation	Was data saturation discussed?	Data saturation was discussed within the research team
23. Transcripts returned	Were transcripts returned to participants for comment and/or correction?	Each transcript was returned to the individual corresponding participant. We received no comments and corrections
**Domain 3: Analysis and findings**		
*Data analysis*		
24. Number of data coders	How many data coders coded the data?	Two (Author 1 and Author 2)
25. Description of the coding list	Did authors provide a description of the coding list?	The coding list is described in Data analysis part of the Methods section
26. Derivation of themes	Were themes identified in advance or derived from the data?	Themes were identified in advance, but we also made use of a code called ‘other’ in which relevant other themes were categorized. This is in line with the coding reliability approach to thematic analysis
27. Software	What software, if applicable, was used to manage the data?	Microsoft Word and Microsoft Excel
28. Participant checking	Did participants provide feedback on the findings?	We asked the participants to reflect on the findings of the study and received no comments or corrections. The participants mentioned that they recognized the modular way of working
*Reporting*		
29. Quotations presented	Were participant quotations presented to illustrate the themes/findings? Was each quotation identified?	We used various quotations from our participants. Moreover, we created multiple figures to illustrate our results
30. Data and findings consistent	Was there consistency between the data presented and the findings?	We present an analytic story where we highlight the two key themes of the study
31. Clarity of major themes	Were major themes clearly presented in the findings?	We present two major themes in the Results section
32. Clarity of minor themes	Is there a description of diverse cases or discussion of minor themes?	The description of the diverse outpatient care teams are presented in the Results section of the study. Minor themes are presented in the running text as part of each major theme

## Appendix 2. Topic list

1. General questions
  - What is your name and age?
  - Can you briefly describe your current job and your role and responsibilities in the outpatient care provision for children with anorexia nervosa?
2. Modular perspective on outpatient care for children with anorexia nervosa
  - Could you please elaborate on the healthcare professionals/organizations involved in the outpatient care for children with anorexia nervosa?
  - Can you describe the specific roles and responsibilities of these healthcare professionals and organizations?
  - How is the intake process for new patients organized?
  - How do you deal with situations when there is insufficient capacity at the hospital?
  - What is your perspective on the practical implementation of this intake process?
  - What communication channels exist among the healthcare professionals?
  - To what extent is the pediatrician the main point of contact, or do the parties involved also have direct communication with one another?
  - To what extent does each patient receive the same treatment plan?
  - To what extent do patients and their parents/representatives have any influence or say in shaping the treatment plan?
3. Continuity of care
  - To what extent do you ever experience difficulties with accessing the patient's medical history (both within and outside the hospital)?
  - To what extent is patient information shared among the various healthcare professionals and organizations involved?
  - What is your assessment of the quality and availability of patient information?
  - How can the availability of information be improved, if necessary?
  - Do you perceive any duplication of data collection at times?
  - To what extent is a patient consistently treated by the same physician and does it vary from one visit to another?
  - To what extent is it preferable if multiple professionals are involved in outpatient care for children with anorexia nervosa?
  - To what extent is it problematic if multiple professionals are involved in outpatient care for children with anorexia nervosa?
  - To what extent is there collaboration between the involved healthcare professionals and organizations regarding the patient's treatment plan?

## Appendix 3. Structured codebook

**Table:**

**Main category**	**Sub-category**	**Definition**	**Source**	**Example**
Modular care provision	Modules (MO)	All data related to the division of tasks between department and involved ones	Soffers et al. (2014)	“As a pediatrician I always want a dietician and a primary care psychologist to be involved.” “We collaborate with third-line psychiatrist if the cases become too complex for primary care psychologists.”
	Components (CO)	All the data in relation to the activities related to a specific department or specialist	Peters et al. (2020)	“Dieticians calculate the nutritional needs.” “I conduct an electro-cardiogram and blood tests which are regularly assessed.”
	Interfaces (IF)	Data related to interaction and communication between departments and specialists	Peters et al. (2020)	“We conduct a multidisciplinary meeting every two months in which all patients are discussed.” “I received a phone call form one of our primary care psychologists to discuss a patient who possibly had an eating disorder.”
Continuity of care	Informational continuity (IC)	All the data related to the availability of information with regards to patient’s other healthcare encounters	Haggerty et al. (2003)	“Within the hospital’s system we can read each other’s notes through our electronic health record.” “I spoke with the mental healthcare institute employees, and I said that I want to receive the psychosocial information they gather.”
	Management continuity (MC)	The data related to the coordination and organization of provided care as experienced by patients	Haggerty et al. (2003)	“We always try to do some form of shared-decision-making. But patients find it difficult because they don’t have an overview of the available possibilities.” “The eating list is put together with the patients and parents. This completely customized.”
	Relational continuity (RC)	Data related to the relationship and familiarity between care provider and receiver	Haggerty et al. (2003)	“We actually always succeed in linking the same pediatrician to the same patient.” “But if you invest time in a first consultation, you will see that the relationship between pediatrician and patient changes positively, which can be beneficial later on.”
